# Sociodemographic Determinants of Telemedicine Uptake Among Adults in the United States

**DOI:** 10.7759/cureus.103972

**Published:** 2026-02-20

**Authors:** Emmanuel A Mensah, Afia A Otuo, Enoch Twum-Damoah, Evans Y Peprah, Nelson A Bronya, Sheilla Jebiwot, Oluseyi A Aderinwale, Omodele S Francis, Derrick N Owusu

**Affiliations:** 1 Department of Epidemiology and Biostatistics, College of Public Health, East Tennessee State University, Johnson City, USA; 2 Municipal Health Directorate, Ghana Health Service, Sunyani, GHA; 3 Public Health Department, Central Michigan University College of Medicine, Mount Pleasant, USA; 4 Lower Manya Krobo District Health Directorate, Ghana Health Service, Odumase Krobo, GHA; 5 Deparment of Geography, Environment and Sustainability, University of North Carolina, Greensboro, USA; 6 Department of Epidemiology and Biostatistics, College of Public Health, East Tennessee State University, Johnson city, USA

**Keywords:** nhanes data, sociodemographic factors, survey logistic regression, telehealth utilization, telemedicine (tm)

## Abstract

Introduction: Recent technological advancements have made telemedicine a potent alternative to in-person hospital care. However, there are limited nationwide population-based estimates of the utilization of telemedicine, and the adoption of these innovative interventions may vary among sociodemographic groups. We conducted a nationally representative study to determine the rate of telemedicine use and to identify the sociodemographic factors associated with usage in the United States.

Methods: This study included 7797 respondents aged 20 years and above from the National Health and Nutrition Examination Survey (NHANES). The survey period for the data used was August 2021 to August 2023. Statistical analysis included frequency distribution and the Rao-Scott chi-square test to determine factors associated with telemedicine use. Factors associated with telemedicine use at a statistical significance level of 0.05 were further modeled using bivariate and multivariate survey logistic regression.

Results: The use of telemedicine among adults was 2694 (32.46%), with a 95% confidence interval ranging from 31.22% to 33.71%. The factors associated with increased odds of telemedicine use were being female (AOR = 1.47, 95% CI: 1.29-1.67), having a college education or associate degree (AOR = 1.58, 95% CI: 1.34-1.86), having higher educational attainment (AOR = 2.02, 95% CI: 1.70-2.39), and having poor general health (AOR = 1.66, 95% CI: 1.41-1.95). The factors associated with reduced odds of telemedicine use were being uninsured (AOR = 0.37, 95% CI: 0.27-0.51) and not having a health facility that an individual usually visits (AOR = 0.42, 95% CI: 0.33-0.54).

Conclusion: Although telemedicine use appears to have declined slightly since the pandemic peak, the prevalence reported in this study confirms that telemedicine remains a sustained and routine component of healthcare delivery even in the post-pandemic era. The development of public health policies and interventions may focus on promoting usage among males and improving the convenience of use among individuals with low digital literacy.

## Introduction

Healthcare access remains a significant challenge in developing and developed countries [1]. However, recent technological advancements have made telemedicine a potent alternative to in-person hospital care [2,3]. The United States Institute of Medicine defines telemedicine as the use of electronic information and communication technology to assist and deliver medical care when the client or patient and the healthcare practitioner are separated by distance [4]. The concept encompasses simple processes such as health education through ordinary telephone use and complex procedures such as telesurgery [4]. In addition to improving access to healthcare, the implementation of telemedicine results in client satisfaction, high convenience, and high-quality care while minimizing waiting time, reducing costs, and lowering the risk of nosocomial infection [5-7].

In the United States, a Medicare report indicated that the number of telemedicine visits increased from 860,807 in 2019 to 53,084,788 in 2020 and 37,047,663 in 2021. These visits accounted for 0.08%, 5.3%, and 3.5% of the total Medicare visits for 2019, 2020, and 2021, respectively [8]. Among adults, surveys have indicated that the prevalence of telemedicine use in the past 12 months of the survey was 37% in 2021 and 43% in 2022 [3,9]. Despite the marginal gains in telemedicine usage shown in the Medicare and survey reports, some administrative and individual obstacles hinder maximum integration into healthcare. Administrative issues that prevent widespread use include adverse payment schemes, regulatory and policy obstacles, a lack of incentive to adopt alternatives to in-person treatment, and concerns regarding information privacy and data security [5,6,10]. Individual-level barriers include internet and technology access, digital literacy, defined as the knowledge and ability necessary to use telehealth devices, and a lack of motivation to use these devices due to poor perceptions [3,10-12].

The purpose of this study was to determine the rate of telemedicine use and to identify the sociodemographic factors associated with usage. This has become necessary for several reasons. First, there are limited nationwide population-based estimates of the utilization of telemedicine [9,13]. The use of telemedicine gained prominence during the COVID-19 pandemic because of lockdowns and infection control measures. It was then scientifically predicted that it would remain a critical aspect of medical treatment [2,14]. To guarantee fair uptake and equal accessibility, variations in telemedicine utilization among social groups must be estimated continuously. Second, the scientific literature presents diverse views on the relationships between sociodemographic characteristics and telemedicine use. While some studies reported that significant variations were not observed by key sociodemographic characteristics, such as household income, race, and education [3,15], other findings suggest that disparities were observed on the basis of sociodemographic differences [9]. The different views portrayed in the available literature suggest that the relationship between telemedicine use and sociodemographic characteristics depends on the subpopulations or geographic areas being studied. It is therefore justifiable to use a nationally representative sample to identify the determinants of telemedicine use in the United States. Finally, innovative interventions to provide medical resources remotely are being developed and implemented rapidly. However, comprehensive assessments guiding strategic policy planning are lacking [16]. Studies on telemedicine use and its associated sociodemographic factors will be vital for intervention and public health policy development to ensure equitable and universal access among subpopulations [17]. This work will also contribute to the growing literature on telemedicine in the post-COVID-19 era. We hypothesized that telemedicine adoption is unequal among sociodemographic groups.

## Materials and methods

Study design, data sources, and variables

This was a cross-sectional study of the utilization of remote healthcare delivery and its relationship to demographic characteristics such as sex, race, marital status, educational attainment, and age. Other factors included in the study were the insurance status of the respondent, family poverty level index, perceived general health, and facility for usual healthcare visits. Data for the study were sourced from the National Health and Nutrition Examination Survey (NHANES), previously known as the National Health Examination Survey [18]. NHANES, a regular survey initiated by the National Center for Health Statistics, Centers for Disease Control and Prevention, provides information on the frequency of illnesses with significant public health implications and the risk factors linked to them, tracking disease trends and exposure to the environment. Another purpose of the survey is to investigate emerging public health needs, such as telehealth usage, and to maintain countrywide representative baseline data on health indicators.

NHANES uses a multistage probability sampling design to produce a nationally representative sample. In the first stage, primary sampling units consisting of counties or groups of counties are selected. The second stage involves choosing smaller geographic segments, neighborhoods, within these units, followed by the random selection of households within each segment. Finally, individuals within selected households are randomly chosen according to demographic characteristics, including age, sex, and race/ethnicity. In the selected households, eligible respondents were aged between 20 and 59 years. In addition, information is collected on all people aged 0-19 years and those aged 60 years and above in the households. For households with up to three eligible individuals, one person was interviewed at random, whereas two persons were selected from households with four or more eligible individuals.

The outcome variable, "Telemedicine Use," was assessed with the question, "In the past 12 months, have you had an appointment with a doctor, nurse, or other health professional by video conference or by phone?" The measurement of the independent variables is specified in Table 1.

**Table 1:** List and definitions of NHANES variables considered potential sociodemographic determinants of telemedicine use among adults in the United States NHANES:National Health and Nutrition Examination Survey.

NHANES Variable	Characteristics	Definition/Measurement
RIAGENDR	Sex	Gender of the participant
RIDRETH1	Race	Recode of reported race and Hispanic origin information of respondent
DMDEDUC2	Educational level	Highest grade or level of school completed or the highest degree received
RIDAGEYR	Age	Age in years of the participant at the time of screening
DMDMARTZ	Marital status	Marital status
HIQ011	Insurance status	Respondent covered by health insurance or some other kind of health care plan? [Include health insurance obtained through employment or purchased directly as well as government programs like Medicare and Medicaid that provide medical care or help pay medical bills.]
INDFMMPC	Family poverty level index	Family monthly poverty level index, a ratio of monthly family income to the HHS poverty guidelines specific to family size
HUQ010	Perceived general health	“Would you say your health in general is Excellent, Very good, Good, Fair or Poor”
HUQ030	Routine healthcare facility	A place respondent usually go/goes when he/she is sick or needs advice about health

Study population

The study involved civilians aged 20 years and above in the contiguous United States. Persons living in institutional group quarters and active-duty military personnel were excluded from the survey. During the study period, 11,933 participants were interviewed. After individuals under 20 years of age and those who did not respond were excluded, 7797 observations remained. This number served as the sample size for the study.

Data preparation and statistical analysis

The NHANES dataset was available in segments. The variables used in this work were from the demographic data files, and three data files were from the questionnaire data. The data files downloaded were hospital utilization and access to care, income, and health insurance. The downloaded datasets were imported into SAS and merged by sequence number. The variable age was initially measured on a continuous numerical scale from 0 to 80. For the purposes of this analysis, age was categorized as 0-19 years, observations deleted, 20-44 years, 45-64 years, and 65 years and above. Education was recoded from eight response categories to three response levels by combining "Less than 9th grade," "9-11th grade (Includes 12th grade with no diploma)," and "High school graduate/GED or equivalent" into "High School and Below." The categories "Some college or AA degree" and "College graduate or above" remained unchanged. The responses "refused," "don’t know," and "missing" were all classified as missing (.). For the variable race, "Mexican American" and "Other Hispanic" were initially presented separately. These two race categories were combined. For the remaining variables, no major changes were made, except for changing the responses "refused" and "don’t know" to "missing" (.). Following data cleaning and recoding, a frequency distribution for all variables was determined. A Rao Scott chi-square test was performed to determine factors associated with telemedicine use. Factors associated with telemedicine use at a statistical significance level of 0.05 were further modeled using survey-weighted bivariate and multivariate logistic regression. From that analysis, the crude odds ratio (COR), adjusted odds ratio (AOR), confidence interval, and p values were reported.

## Results

Background characteristics and telemedicine use

As shown in Table 2, the majority of the respondents were female, 4319 (51.57%). Among the racial and ethnic groups, non-Hispanic White individuals, 4547 (59.97%), constituted the majority. A total of 1323 (16.69%) of the respondents were Mexican American or other Hispanic, 994 (11.06%) were Black, and 933 (12.27%) were all other races, including individuals with multiracial backgrounds. Most of the respondents, 2786 (36.60%), had high school qualifications and below, whereas 2621 (33.97%) had college degrees. A significant proportion of the respondents were between the ages of 20 and 44 years, 2663 (44.83%), and were married or living with their partners, 4132 (60.30%). Only a few, 667 (9.54%), of the respondents did not have health insurance coverage, and the majority of the respondents, 4098 (64.72%), were categorized as having high family income status. Most of the respondents, 6262 (83.25%), perceived their general health as good, and 6860 (85.56%) had a health facility that they usually visit. The usage of telemedicine among adults was 2694 (32.46%), with a 95% confidence interval of 31.22% to 33.71%. 

**Table 2:** Sociodemographic characteristics of the study respondents, NHANES August 2021 to August 2023 NHANES: National Health and Nutrition Examination Survey.

Characteristics	Frequency (N=7797)	Weighted Percentage % (95%CI)
Sex		
Male	3478	48.43 (47.07–49.78)
Female	4319	51.57 (50.22–52.93)
Race		
Mexican American/Hispanic	1323	16.69 (15.68–17.71)
Non-Hispanic White	4547	59.97 (58.65–61.30)
Non-Hispanic Black	994	11.06 (10.25–11.87)
Multiracial/other races	933	12.27 (11.37–13.18)
Educational level		
High school and below	2786	36.60 (35.29–37.92)
College/associate degree	2364	29.42 (28.20–30.64)
College graduate & above	2621	33.97 (32.70–35.25)
Age		
20–44	2663	44.83 (43.46–46.20)
45–64	2553	32.71 (31.45–33.98)
65+	2581	22.46 (21.52–23.40)
Marital status		
Married/living with partner	4132	60.30 (59.00–61.59)
Widowed/divorced/separated	2019	19.01 (18.08–19.94)
Never married	1620	20.70 (19.59–21.81)
Insurance status		
Insured	7097	90.46 (89.62–91.30)
Uninsured	667	9.54 (8.70–10.38)
Family poverty level index		
Low Income	1661	21.58 (20.43–22.74)
Middle Income	936	13.69 (12.69–14.70)
High Income	4098	64.72 (63.35–66.10)
Perceived general health		
Good	6262	83.25 (82.30–84.20)
Poor	1532	16.75 (15.80–17.70)
Routine healthcare facility		
Yes	6860	85.56 (84.53–86.60)
No	933	14.44 (13.40–15.47)
Telehealth use		
No	5103	67.54 (66.29–68.78)
Yes	2694	32.46 (31.22–33.71)

Factors associated with telemedicine use

The Rao-Scott chi-square test of association (Table 3) revealed that all the factors under consideration were associated with telemedicine use. These variables include sex (χ^2^ = 59.67, p < 0.0001), race (χ^2^ = 23.63, p < 0.0001), educational level (χ^2^ = 113.09, p < 0.0001), age (χ^2^ = 24.31, p < 0.0001), and marital status (χ^2^ = 18.98, p < 0.0001). Other factors associated with telemedicine use according to the chi-square test were insurance status (χ^2^ = 134.15, p < 0.0001), family poverty level index (χ^2^ = 22.34, p < 0.0001), perceived general health (χ^2^ = 23.27, p < 0.0001), and routine healthcare facilities (χ^2^ = 131.27, p < 0.0001).

**Table 3:** Chi-square test of the associations of factors associated with telehealth use among adults in the United States, NHANES August 2021 to August 2023 NHANES: National Health and Nutrition Examination Survey.

Characteristics	Telehealth Use				χ^2^	P-value
	No		Yes			
	Frequency	Percentage	Frequency	Percentage		
Sex						
Male	2435	72.61	1043	27.39	59.67	<0.0001
Female	2668	62.78	1651	37.22		
Race						
Mexican American/Hispanic	942	73.77	381	26.23	23.63	<0.0001
Non-Hispanic White	2891	65.52	1656	34.48		
Non-Hispanic Black	663	67.28	331	32.72		
Multiracial/other races	607	69.14	326	30.86		
Educational level						
High school and below	2050	75.86	736	24.14	113.09	<0.0001
College/associate degree	1488	65.56	876	34.44		
College graduate & above	1542	60.10	1079	39.90		
Age						
20–44	1836	70.79	827	29.21	24.31	<0.0001
45–64	1637	65.43	916	34.57		
65+	1630	64.10	951	35.90		
Marital status						
Married/living with partner	2676	66.50	1456	33.50	18.98	<0.0001
Widowed/divorced/separated	1290	65.28	729	34.72		
Never married	1119	72.57	501	27.43		
Insurance status						
Insured	4494	65.17	2603	34.83	134.15	<0.0001
Uninsured	579	88.97	88	11.03		
Family poverty level index						
Low income	1137	71.84	524	28.16	22.34	<0.0001
Middle income	623	69.18	313	30.82		
High income	2567	64.33	1531	35.67		
Perceived general health						
Good	4209	68.87	2053	31.13	23.27	<0.0001
Poor	894	61.15	638	38.85		
Routine healthcare facility						
Yes	4320	64.42	2540	35.58	131.27	<0.0001
No	780	85.93	153	14.07		

The final adjusted model (Table 4) revealed that females were 47% (AOR = 1.47, 95% CI: 1.29-1.67) more likely to use telemedicine than males were. Individuals who had some college education or an associate degree were 1.58 (AOR = 1.58, 95% CI: 1.34-1.86) times more likely to use telemedicine than those with a high school education or fewer qualifications. Furthermore, college graduates with higher educational attainment were 2.02 (AOR = 2.02, 95% CI: 1.70-2.39) times more likely to use telemedicine than those with a high school education or fewer qualifications. Uninsured individuals were 63% (AOR = 0.37, 95% CI: 0.27-0.51) less likely to use telemedicine than those who were insured. Among those who described their general health as poor, 66% (AOR = 1.66, 95% CI: 1.41-1.95) used telemedicine more often than those who described their general health as good. Not having a health facility that an individual usually visits reduced telemedicine use by 0.42 times (AOR = 0.42, 95% CI: 0.33-0.54). Notably, race, age, and family poverty level were not significantly associated with telemedicine use after controlling for other demographic factors.

**Table 4:** Logistic regression of the association of factors associated with telehealth use among adults in the United States, NHANES August 2021 to August 2023 NHANES: National Health and Nutrition Examination Survey, COR: crude odds ratio, AOR: adjusted odds ratio, CI: confidence interval.

Characteristics	COR (95%CI)	P-value	AOR (95%CI)	P-value
Sex				
Male	1.00		1.00	
Female	1.57 (1.40–1.76)	< .0001	1.47 (1.29–1.67)	< .0001
Race				
Non-Hispanic White	1.00		1.00	
Mexican American/Hispanic	0.68 (0.57–0.80)	< .0001	1.00 (0.83–1.22)	0.9789
Non-Hispanic Black	0.92 (0.77–1.11)	0.3901	1.03 (0.84–1.26)	0.7989
Multiracial/other races	0.85 (0.71–1.01)	0.0684	0.92 (0.75–1.13)	0.4125
Educational level				
High school and below	1.00		1.00	
College/associate degree	1.65 (1.43–1.91)	< .0001	1.58 (1.34–1.86)	< .0001
College graduate & above	2.09 (1.82–2.40)	< .0001	2.02 (1.70–2.39)	< .0001
Age				
20-44	1.00		1.00	
45-64	1.28 (1.12–1.47)	0.0004	1.11 (0.95–1.30)	0.2045
65+	1.36 (1.19–1.55)	< .0001	1.08 (0.92–1.27)	0.3676
Marital status				
Married/living with partner	1.00		1.00	
Widowed/divorced/separated	1.06 (0.93–1.21)	0.4205	1.03 (0.89–1.20)	0.6838
Never married	0.75 (0.65–0.87)	0.0002	0.94 (0.79–1.13)	0.5061
Insurance status				
Insured	1.00		1.00	
Uninsured	0.23 (0.18–0.30)	< .0001	0.37 (0.27–0.51)	< .0001
Family poverty level index				
Low income	1.00		1.00	
Middle income	1.14 (0.92–1.41)	0.2362	1.11 (0.89–1.38)	0.3682
High income	1.42 (1.22–1.64)	< .0001	1.10 (0.93–1.31)	0.2661
Perceived general health				
Good	1.00		1.00	
Poor	1.41 (1.22–1.61)	< .0001	1.66 (1.41–1.95)	< .0001
Routine healthcare facility				
Yes	1.00		1.00	
No	0.30 (0.24–0.37)	< .0001	0.42 (0.33–0.54)	< .0001

## Discussion

The purpose of our study was to determine the sociodemographic determinants associated with telemedicine utilization among adults in the United States. Approximately 32% of U.S. adults reported using telemedicine within the past 12 months. This prevalence is slightly lower than estimates reported in national surveys following the peak of the COVID-19 pandemic, where telemedicine utilization ranged between 37% in 2021 and 43% in 2022 [3,9]. Differences in prevalence estimates may be explained by variations in data sources, study periods, and target populations. While earlier studies relied largely on rapid-response surveys such as the Health Information National Trends Survey (HINTS) [3], the current study used NHANES 2021-2023 data, which employ in-person interviews and a complex probability sampling design. Although telemedicine use appears to have declined slightly from pandemic peaks, the prevalence observed in this study confirms that telemedicine remains a sustained and routine component of healthcare delivery in the post-pandemic era.

The present study revealed that sex, educational attainment, insurance status, perceived general health, and having a routine healthcare facility were significantly associated with telemedicine use, whereas race, age, and family poverty level were not significant after adjustment. Compared with males, females were 47% more likely to use telemedicine. This finding is consistent with prior research demonstrating that women generally have greater healthcare utilization patterns and greater engagement with preventive and outpatient services [19]. Women are also more likely to manage health needs for themselves and their families, which may increase exposure to telemedicine platforms.

Educational attainment was strongly associated with telemedicine utilization. Adults with some college education or an associate degree and those with a college degree were significantly more likely to use telemedicine than individuals with a high school education or less. This finding supports existing evidence that digital literacy and health system navigation skills play a central role in the adoption of telehealth services [20]. Even when financial access is available, individuals with limited education may lack the technological skills needed to effectively engage with telemedicine platforms, including video conferencing tools, patient portals, and remote monitoring technologies [21]. This emphasizes education as a structural determinant of digital health equity.

Insurance status emerged as one of the determinants of telemedicine utilization in the present study. Adults without health insurance were 63% less likely to use telemedicine than those who were insured. This finding indicates that, despite being delivered remotely, telemedicine still depends heavily on the traditional healthcare payment system, where insurance coverage determines access to services. Uninsured individuals may face limited access to participating providers, concerns about out-of-pocket costs, and a lack of reimbursement options, all of which can discourage the use of virtual care. As a result, telemedicine may not yet function as a true equal-access alternative for uninsured populations but instead reflects existing gaps in healthcare coverage.

Respondents who perceived their general health as poor were 66% more likely to use telemedicine than those who reported good health. This likely reflects increased clinical demand among individuals managing chronic illnesses, disabilities, or frequent symptoms. Telemedicine may offer a practical alternative for these individuals by reducing the need for transportation, minimizing physical burden, and allowing more frequent follow-up care. This finding supports prior evidence that telemedicine is most utilized by patients with greater health needs [7].

Additionally, having a routine healthcare facility was strongly associated with telemedicine use. Individuals without a usual source of care were significantly less likely to use telemedicine. This suggests that telemedicine functions primarily as an extension of established healthcare relationships rather than a substitute for entry into the healthcare system. Patients who lack a consistent place for care may face barriers not only to in-person services but also to virtual care, further compounding access disparities.

Strengths and limitations

This study used NHANES, a large, nationally representative dataset of the U.S. civilian noninstitutionalized population, which strengthens the generalizability of the findings. The application of survey-weighted analyses and multivariable modeling enhances internal validity and ensures that estimates appropriately reflect national population patterns. The study also incorporated a wide range of sociodemographic and health-related variables, allowing for a comprehensive assessment of telemedicine determinants. Despite these strengths, several limitations must be acknowledged. The study relied on self-reported telemedicine use, which may be subject to recall bias and social desirability bias. The cross-sectional design limits causal inference, and the associations observed cannot be interpreted as temporal relationships. Finally, the NHANES dataset does not include direct measures of internet access, broadband availability, device ownership, or digital literacy, which are critical factors influencing telemedicine utilization.

## Conclusions

Only a marginal decline has been observed in the use of telemedicine from its peak rates during the COVID-19 pandemic. This study revealed that the use of telemedicine may be particularly useful for individuals with poor general health and increased clinical demands, such as individuals with chronic illness, disabilities, and frequent or recurrent symptoms. However, telemedicine usage among males, those who are less educated, and the uninsured remains a challenge. Again, it appears that telemedicine has not yet been fully positioned as an independent avenue for medical care but rather as an extension of healthcare facilities, thereby hindering access to telemedicine for individuals with no affiliations. The development of public health policies and interventions may focus on enhancing the autonomous advancement of telemedicine health practices, promoting usage among males, improving the convenience and user-friendliness among individuals with low digital literacy, and ensuring telemedicine affordability among persons who are uninsured.
